# Redox-Guided DNA Scanning by the Dynamic Repair Enzyme Endonuclease III

**DOI:** 10.1021/acs.biochem.4c00621

**Published:** 2025-02-04

**Authors:** Ayaz Hassan, Filipe C. D. A. Lima, Frank N. Crespilho

**Affiliations:** † São Carlos Institute of Chemistry, University of São Paulo (USP), São Carlos, SP 13566-590, Brazil; ‡ IRCBM, COMSATS University Islamabad (CUI), 1.5 KM Defence Road Off Raiwand Road, Lahore 54000, Pakistan; § Federal Institute of Education, Science, and Technology of São Paulo, Matão, SP15991-502, Brazil

## Abstract

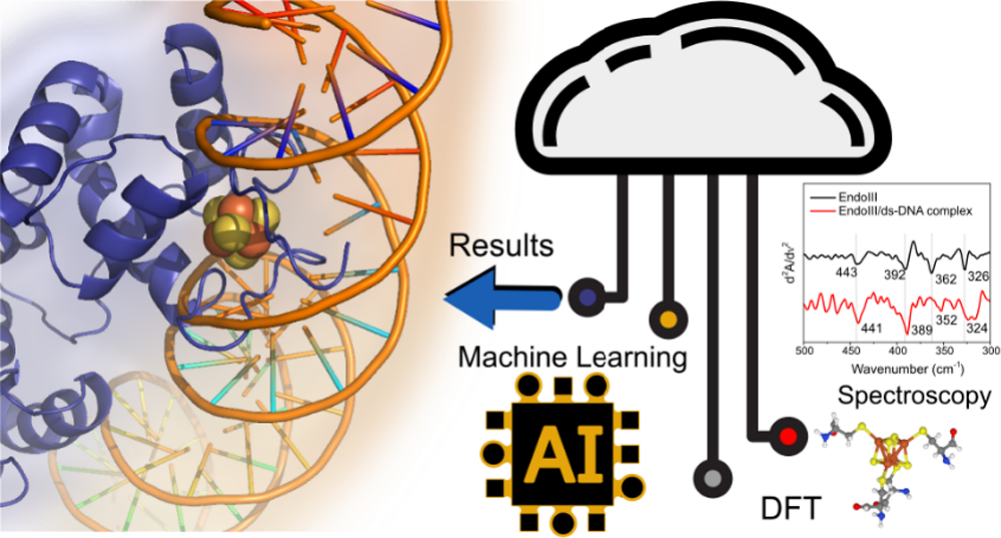

Endonuclease III (EndoIII), a key enzyme in the base excision repair (BER) pathway, contains a [4Fe4S] cluster that facilitates DNA repair through DNA-mediated charge transfer. Recent findings indicate that the redox state of this cluster influences EndoIII’s binding affinity for DNA, modulating the enzyme’s activity. In this study, we investigated the structural and electronic changes of the [4Fe4S] cluster upon binding to double-stranded DNA (dsDNA) using Fourier transform infrared spectroscopy, density functional theory calculations, and machine learning models. Our results reveal shifts in Fe–S bond vibrational modes, suggesting stabilization of the oxidized [4Fe4S] cluster in proximity to negatively charged DNA. A machine learning model, trained on the spectral features of the EndoIII/DNA complex, predicted the enzyme-DNA binding distance, providing further insights into the structural changes upon binding. We correlated the electrochemical stabilization potential of 150 mV in the [4Fe4S] cluster with the enzyme’s DNA-binding properties, demonstrating how the cluster’s redox state plays a crucial role in both structural stability and DNA repair.

## Introduction

Iron–sulfur (Fe–S) cluster-containing proteins are essential for various biological processes, including electron transfer, DNA replication, and DNA repair.^1−4^ These proteins utilize [4Fe4S] clusters as redox cofactors, enabling them to function as molecular switches that modulate key enzymatic activities.^5−7^ Endonuclease III (EndoIII, UniProtP0AB83) is a key enzyme in the base excision repair (BER) pathway, responsible for identifying and excising damaged DNA bases.^8−10^ Central to its function is the [4Fe4S] cluster, which not only maintains the structural integrity of the enzyme but also facilitates redox signaling.^11,12^ Through DNA-mediated charge transfer (DNA-CT), this cluster enables EndoIII to detect and respond to damage within the DNA duplex, transforming a traditionally structural cofactor into a dynamic sensor of DNA integrity.^13^

Similar to other Fe–S cluster-containing enzymes involved in DNA processes, such as DNA primase in eukaryotic DNA replication,^14^ EndoIII’s [4Fe4S] cluster functions as a reversible redox switch.^15^ In its oxidized state ([4Fe4S]^3+^), the cluster enhances EndoIII’s affinity for DNA, enabling efficient electron transfer along the DNA duplex to scan for lesions.^16^ Upon reduction to the ([4Fe4S]^2+^) state, the enzyme dissociates from DNA, facilitating continued scanning across the genome. This redox-driven modulation of DNA binding is integral to the DNA-CT mechanism, wherein disruptions in charge flow signal the presence of DNA damage, triggering repair processes.^8,17^ Such redox signals provide an efficient mechanism for the long-range communication between proteins and DNA. A parallel process is observed in DNA primase during replication, where its [4Fe4S] cluster orchestrates primer synthesis and after polymerase α,^18^ highlighting the versatile role of Fe–S clusters in coordinating DNA-related activities.

**Figure 1:**
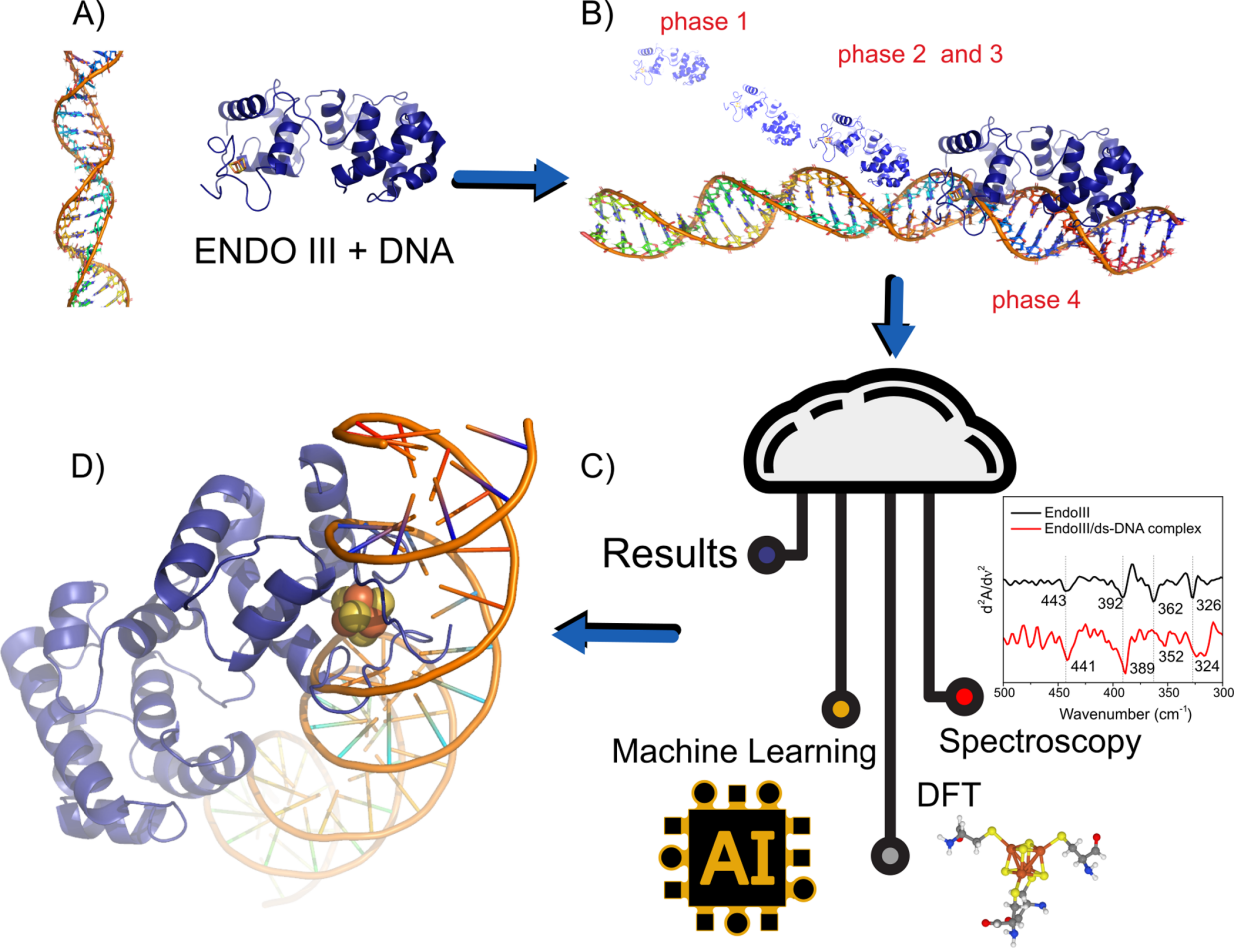
Mechanistic exploration of EndoIII and dsDNA interaction phases integrating machine learning and spectroscopy analysis. (A) Structural depiction of EndoIII and dsDNA complex formation. (B) Sequential phases of interaction between EndoIII and DNA, highlighting the four stages of engagement: phase 1 (initial contact), phases 2 and 3 (progression and deeper interaction), and phase 4 (final binding). (C) Schematic showing the integration of machine learning and spectroscopy data for analyzing the EndoIII and DNA interaction. (D) Concept illustration of the system ENDOIII+DNA. Entropic forces and potential-driven scanning processes are illustrated, contributing to the understanding of binding dynamics through advanced AI-driven and computational modeling approaches.

Despite growing recognition of the [4Fe4S] cluster’s role in DNA-CT, the molecular mechanisms underlying its redox-driven behavior, particularly in EndoIII, remain to be fully elucidated. A deeper understanding of how the cluster’s geometry and electronic structure are altered upon DNA binding and how these changes influence the enzyme’s function in DNA repair is crucial for advancing Fe–S cluster biochemistry. To address these questions, we employed a multidisciplinary approach combining Fourier transform infrared (FTIR) spectroscopy, density functional theory (DFT) calculations, and machine learning models (Figure 1). These complementary techniques allowed us to investigate the structural and electronic dynamics of the [4Fe4S] cluster in EndoIII, particularly how DNA binding alters the vibrational properties and covalency of the Fe–S bonds. This study addresses three key questions: How do the structural and redox properties of the [4Fe4S] cluster change upon DNA binding? Can machine learning accurately predict binding distances and uncover molecular interactions from spectroscopic data? What thermodynamic forces drive the interaction between EndoIII and DNA, and how do they impact the enzyme’s repair function? By addressing these questions, we aim to provide a comprehensive understanding of the interplay among redox regulation, structural dynamics, and DNA binding in EndoIII. This work also explores the broader role of [4Fe4S] clusters in DNA charge transport and repair pathways. We focus on how interactions with DNA alter the vibrational frequencies of Fe–S bonds, providing insights into the electronic and structural dynamics of the cluster upon DNA binding.

## Results and Discussion

### Protein–DNA Interaction

**Figure 2:**
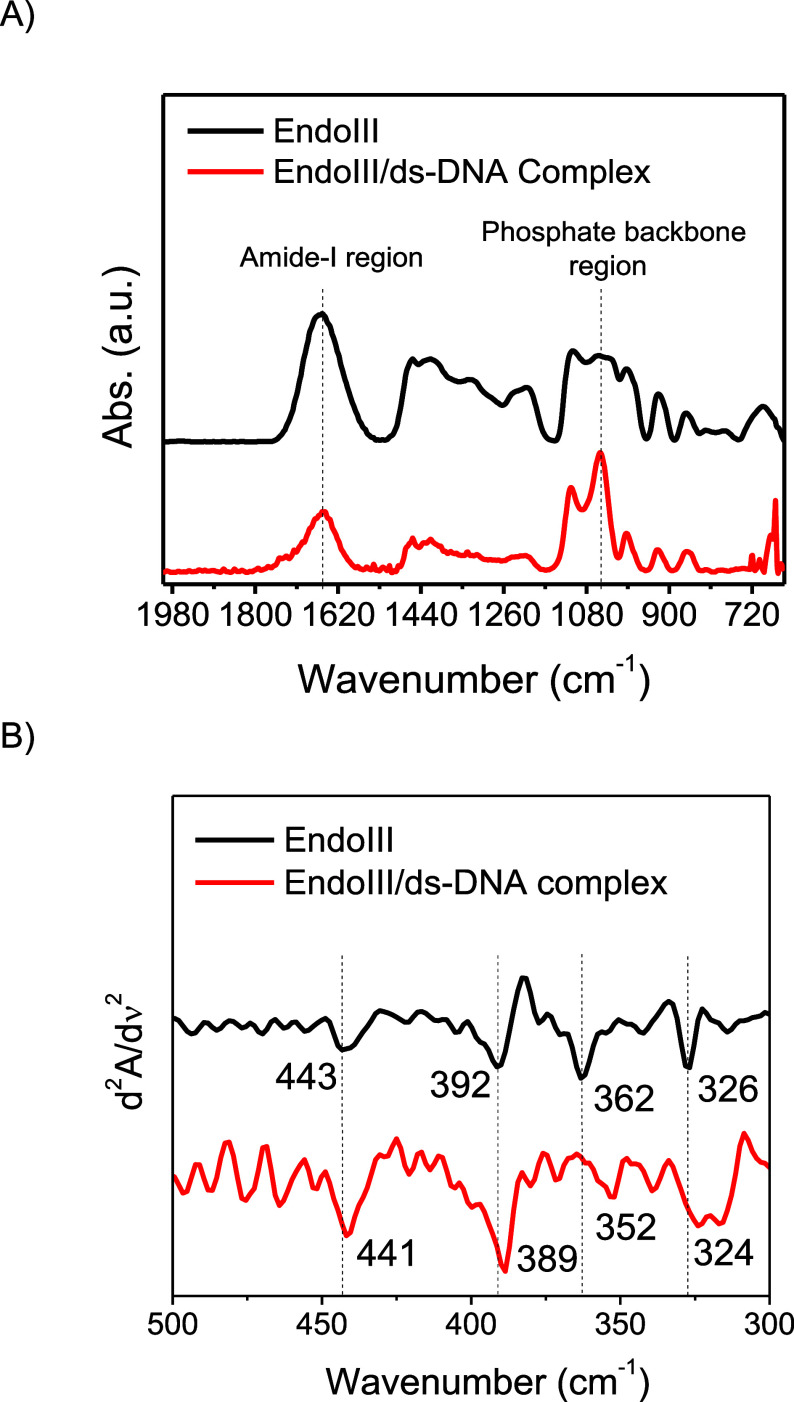
(A) Micro-FTIR spectra recorded in the mid-IR region for EndoIII and EndoIII/dsDNA complex. Thin films of samples were immobilized on a gold-coated glass substrate. Measurements were performed in reflectance mode. (B) Second derivative of Far-IR spectrum of EndoIII (black) and of EndoIII/dsDNA complex (red). Second derivative highlights subtle changes in absorption peaks corresponding to the [4Fe4S] cluster vibrations by enhancing the resolution of overlapping peaks and helps distinguish vibrational modes in the EndoIII-DNA complex.

Spectra in the mid-infrared (mid-IR) region (Figure 2A) revealed key differences between free EndoIII and the EndoIII/dsDNA complex. Notable shifts were observed in the amide-I band (1653 cm^–1^) of the protein, and the phosphate stretching bands (1213 and 1060 cm^–1^) of the DNA backbone were observed upon complex formation. These spectral changes indicate a structural rearrangement in both the protein and DNA during binding. The 3 cm^–1^ downshift of the amide-I band suggests alterations to the secondary structure of EndoIII, likely caused by conformational adjustments upon interaction with the DNA helix. Furthermore, far-infrared (Far-IR) spectra^12^ have provided detailed insights into the behavior of the Fe–S bonds within the [4Fe4S] cluster. To emphasize peak shifts resulting from EndoIII binding to dsDNA, the second derivates of the spectra were analyzed (Figure 2B). In the spectrum of native EndoIII, four distinct peaks corresponding to Fe–S bond vibrations were observed at 443, 392, 362, and 326 cm^–1^. Upon binding to dsDNA, these peaks experienced shifts, particularly the Fe–S (thiolate) bond stretching mode at 362 cm^–1^, which downshifted to 352 cm^–1^. This shift indicates a weakening of the Fe–S bond, which is interpreted as evidence of increased covalency and stabilization of the oxidized [4Fe4S] cluster in the presence of DNA.

DFT calculations supported the experimental findings, predicting comparable shifts in the vibrational frequencies of Fe–S bonds upon DNA binding. The calculated bond lengths for the Fe–S (thiolate) bonds increased slightly in the DNA-bound state, consistent with the experimental observation of bond weakening. These results confirm that the [4Fe4S] cluster undergoes structural changes in the presence of negatively charged DNA, favoring its oxidized state. This structural modulation facilitates the redox communication necessary for DNA repair processes mediated by EndoIII. FTIR spectral analysis revealed significant shifts in key vibrational modes upon formation of the EndoIII/dsDNA complex, reflecting molecular interactions and structural adjustments during binding. Notable shifts were observed in CH out-of-plane bending vibrations (720 and 780 cm^–1^) and the sugar–phosphate backbone (782 cm^–1^), indicating alterations in the DNA backbone’s structure as it interacts with EndoIII. Additionally, the disappearance of the deoxyribose ring vibration (890 cm^–1^) indicates that EndoIII induces conformational changes in the DNA, likely bending or distorting the helix to access damaged bases. These findings align with EndoIII’s known mechanism, wherein the enzyme interacts with the phosphate backbone and bases to identify and excise oxidative damage effectively.

EndoIII/dsDNA interactions involve not only electrostatic interactions but also specific conformational adjustments in both molecules, as evidenced by changes in the vibrational frequencies. The strong correlation between selected FTIR spectral features and the binding distance of EndoIII to dsDNA shows strong associations, indicating that these vibrational changes are closely linked to the spatial orientation of the protein–DNA complex. Peaks associated with the sugar–phosphate backbone, such as the 1213 cm^–1^ phosphate stretching and the CH_2_ wagging at 1329 cm^–1^, exhibit correlations with binding distance. These features suggest that structural changes in the DNA backbone serve as key indicators of the enzyme-DNA proximity. Analysis of specific peaks (780, 1055, 1110, and 1376 cm^–1^) provides further insights into the interaction between EndoIII and dsDNA. The 780 cm^–1^ peak, associated with CH out-of-plane bending, shifts slightly, indicating subtle structural rearrangements in the DNA upon complex with EndoIII. The shift observed in the 1055 cm^–1^ peak, corresponding to phosphate backbone vibrations, suggests a direct interaction with the DNA backbone, confirming EndoIII’s role in altering the DNA’s structure to access damaged regions. Meanwhile, the 1376 cm^–1^ peak, related to cytosine and guanine base vibrations, shows distortion into smaller peaks, indicating specific interactions between EndoIII and these nucleotide bases. This observation aligns with EndoIII’s role in detecting oxidative damage, particularly in guanine-rich regions of the DNA. The analysis of key peaks further reinforces the enzyme’s ability to induce structural alterations in the DNA during the repair process.

### Machine Learning

Taken together, the FTIR analysis, machine learning predictions, and thermodynamic results provide a comprehensive understanding of the interaction between EndoIII and dsDNA. The shifts in FTIR peaks reveal structural adjustments in both the enzyme and DNA during binding, while the correlation between spectral features and the binding distance emphasizes the importance of specific vibrational modes in driving these interactions. The accuracy of the machine learning model highlights the potential of combining experimental data with predictive algorithms to quantify binding distances and to infer molecular interactions. Moreover, the detailed analysis of key FTIR peaks and the binding distance distribution reveals the enzyme’s dynamic behavior during its interaction with DNA, suggesting that additional factors such as redox signaling via the [4Fe4S] cluster could further influence binding affinity and DNA repair efficiency. These insights lay the foundation for future studies on how environmental conditions and redox states modulate EndoIII’s activity, particularly in the context of DNA-mediated charge transfer and redox regulation.

The use of machine learning in this context allows us to explore the molecular fingerprints of the isolated DNA, the EndoIII enzyme, and the DNA/EndoIII complex. By analyzing the spectral changes in specific peak positions and intensities, we can infer the nature of the interactions between the enzyme and DNA. In this study, machine learning models have been employed to predict the binding distance between EndoIII and dsDNA based on IR data. The residual plot shows that the model accurately predicts binding distances, with residuals centered around zero, indicating minimal deviation between predicted and actual values. This suggests that the model generalizes well to unseen data and accurately captures most of the variance in the binding distances. Feature importance analysis highlights the necessity of considering both dsDNA and EndoIII/dsDNA complex features in predicting the binding properties. Changes in both DNA alone and the protein–DNA complex provide relevant information about interaction dynamics.

**Figure 3:**
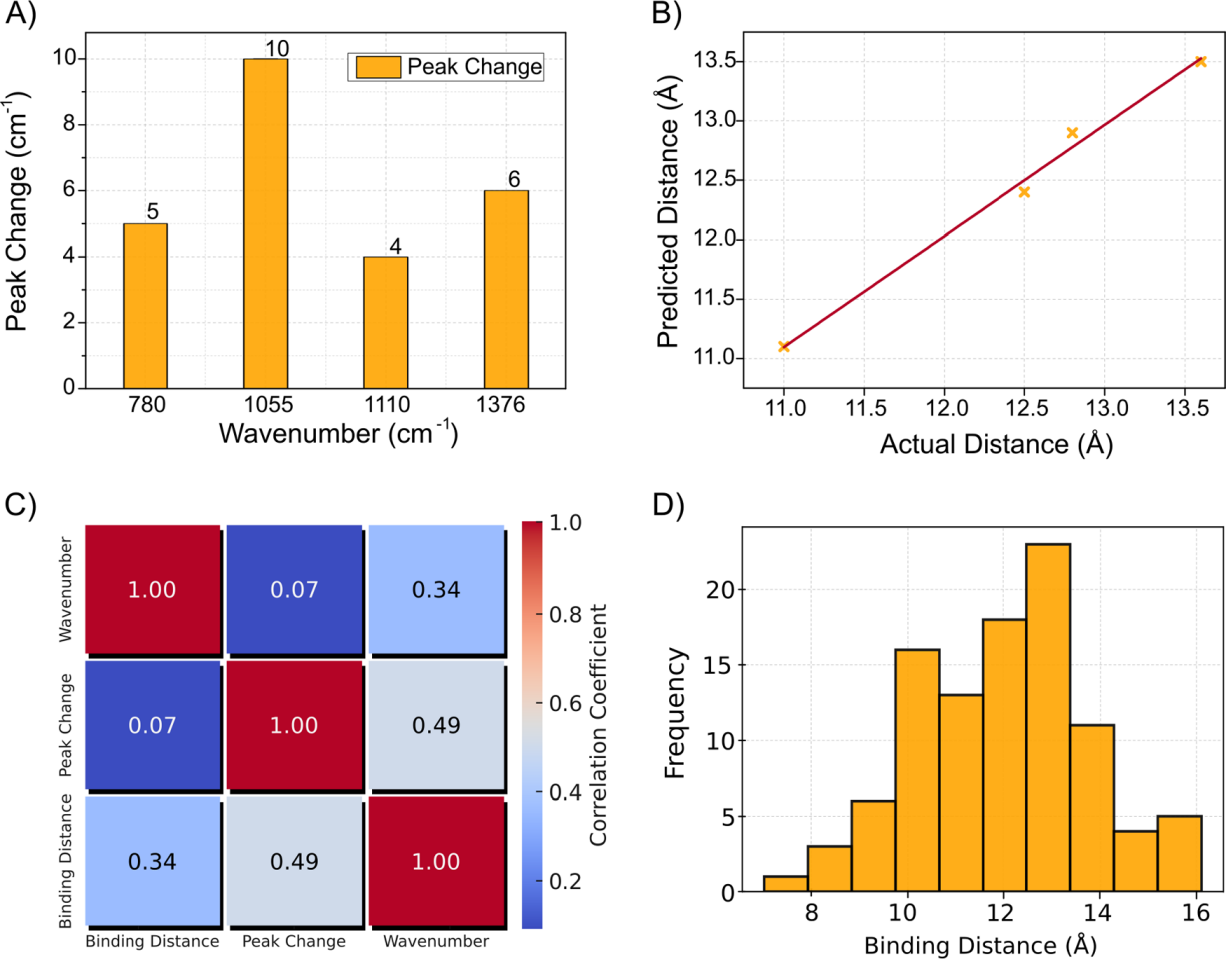
(A) FTIR peak analysis of EndoIII and dsDNA. A comparison of the spectral changes between dsDNA and the EndoIII/dsDNA complex. Bar chart displays key vibrational peaks, highlighting changes in peak intensity and shifts upon DNA binding. (B) Prediction vs actual binding distance (Å). Scatter plot comparing the predicted binding distances to the actual distances (obtained from experimental peak shifts), with the diagonal red dashed line representing perfect correlation. (C) Correlation Heatmap between spectral features and binding distance. Heatmap shows the correlation between the selected FTIR spectral features (from dsDNA and EndoIII/dsDNA complex) and the binding distance of EndoIII to dsDNA. High positive correlation (closer to 1) suggests that the spectral features are associated with the spatial orientation of the enzyme-DNA complex. (D) Histogram of binding distance distribution (Å). Histogram showing the distribution of binding distances between EndoIII and dsDNA in angstroms (Å) predicted by machine learning.

Figure 3 provides an analysis of the molecular interactions between EndoIII and dsDNA through multiple visual representations. In Figure 3A, a bar chart highlights key FTIR vibrational peaks, comparing spectral changes between dsDNA and the EndoIII/dsDNA complex, and focuses on specific FTIR peaks at specific wavenumbers (780, 1055, 1110, and 1376 cm^–1^), showing the interactions between EndoIII and the DNA sugar–phosphate backbone and shifts in peak intensity that reveal molecular interactions. Figure 3B compares predicted binding distances to actual values with a scatter plot demonstrating model accuracy, as points closely follow the red dashed line representing ideal predictions. Figure 3C presents a heatmap illustrating the correlation between selected spectral features and the binding distance of EndoIII to dsDNA, with higher correlations indicating an association between these features and enzyme-DNA spatial orientation. Lastly, Figure 3D shows a histogram of the distribution of binding distances, reflecting the variability in interaction distances across different conditions. The binding distance distribution ranges from 7.0 to 16 Å, reflecting the dynamic nature of EndoIII as it scans for damaged bases along the DNA. The variability in binding distances suggests that the enzyme adopts various orientations relative to the DNA, consistent with its scanning mechanism. Together, these visualizations offer a detailed understanding of the biochemical and structural dynamics of the EndoIII-DNA interaction, supported by FTIR and machine learning analyses. IR spectra offer a detailed view of the vibrational modes of molecular bonds, allowing us to track specific interactions. We analyzed various peaks in the IR spectra corresponding to both DNA and EndoIII and compared them to the DNA/EndoIII complex:

1. *CH Out-of-plane bending vibrations (720 and 780 cm*^*–1*^*):* These peaks are associated with vibrations in the CH bonds of DNA. A notable change was observed in the peak at 780 cm^–1^, where a small shift occurred in the dsDNA spectrum, indicating a slight structural rearrangement in the complex.
2. *Sugar–phosphate vibration (782 cm*^*–1*^*):* The peak at 782 cm^–1^ is attributed to the sugar–phosphate backbone of DNA. A small peak shift was observed in the complex spectrum, suggesting that the backbone structure of the DNA is affected by binding to EndoIII. This is consistent with the known mechanism of EndoIII, which involves interaction with the phosphate backbone to excise damaged bases.
3. *Deoxyribose ring vibration (890 cm*^*–1*^*):* This peak is associated with the deoxyribose sugar in DNA. In the complex, this peak disappears, indicating a strong interaction between EndoIII and the deoxyribose ring. The disappearance of this peak suggests a conformational change in the DNA structure upon binding, possibly related to bending or distortion induced by the enzyme.
4. *O–P–O bending (967 cm*^*–1*^*):* This peak, corresponding to the O–P–O bond in the DNA backbone, also disappears in the complex, further supporting the idea that EndoIII significantly alters the DNA’s backbone structure during binding.
5. *CH*_*2*_*Wagging and N–H deformation (1281, 1329, and 1376 cm*^*–1*^*):* These peaks correspond to CH_2_ wagging vibrations and N–H deformations in both DNA and EndoIII. The shifts and changes in intensity observed in the complex suggest that both the DNA and the protein undergo structural adjustments during the interaction. Notably, the peak at 1376 cm^–1^, associated with C–N stretching in cytosine and guanine, is distorted into several smaller peaks in the complex, indicating that EndoIII interacts specifically with these nucleotide bases.

Using spectral features extracted from IR data, a Ridge regression machine learning model was trained to predict the binding distance between EndoIII and dsDNA. This binding distance is a key parameter that reflects the spatial orientation and proximity of the enzyme to DNA, directly influencing its enzymatic activity. The “actual distances” referenced here were derived from the peak shifts observed in the FTIR analysis, as illustrated in Figure 3A. Two key spectral features were used for prediction: changes in the peak values for dsDNA and EndoIII/dsDNA complex. The regression model was trained using these features to predict the known binding distances, which ranged from 7.0 to 16 Å. The model’s coefficients, displayed in the feature importance plot, reveal the relative contribution of each feature to the final prediction. In this case, both the dsDNA feature and the EndoIII/dsDNA feature had a strong influence on the model’s predictions, indicating that both the isolated DNA and the DNA–protein complex spectra provide critical information about the binding interaction.

**Figure 4:**
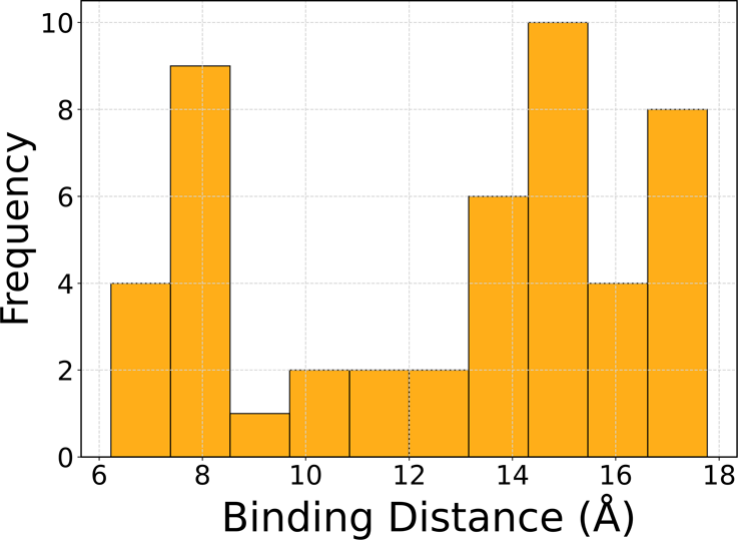
Histogram of 50 Binding Distances (Å) from the 1P59 crystallographic structure.^19^ Histogram illustrates the distribution of selected distances between EndoIII and dsDNA.

The prediction vs actual distance plot shows a correlation between predicted and actual binding distances, with most points lying close to the diagonal reference line. This indicates that the model was able to make accurate predictions for most of the data points, demonstrating the utility of IR features in estimating the spatial orientation of protein–DNA complexes. The correlation heatmap further supports this conclusion, as it demonstrates a correlation between the selected spectral features and the binding distance. The high correlation values between the features and the target variable indicate that the spectral shifts observed in the IR data are linked to the binding geometry of the EndoIII/dsDNA complex. For comparison, we also present selected distances from the 1P59^19^ PDB structure in Figure 4. These include measurements from some of the closest amide groups, the [4Fe–4S] cluster, and the backbone/phosphate groups near the DNA in the X-ray structure (Figure S2). While the histogram does not encompass all possible distance combinations, it effectively represents a similar range of distances predicted by the machine learning model. The machine learning approach achieves comparable results by relying solely on adjustments to the frequency shifts observed in the FTIR spectrum, highlighting its predictive capability.

The mechanism of binding of EndoIII to dsDNA involves a series of structural adjustments in both DNA and the enzyme. As indicated by the IR data, these adjustments are primarily seen in the sugar–phosphate backbone and base-specific interactions. The disappearance of several peaks in the DNA spectrum upon binding (such as those at 890 and 967 cm^–1^) suggests that the enzyme induces significant conformational changes in the DNA structure, likely bending or twisting it to allow access to the damaged base. This interpretation is consistent with previous structural studies that show protein binding to DNA via its active site, which interacts directly with the phosphate backbone and the base pairs.^20^ The enzyme’s binding pocket is positioned such that it can excise the damaged base while maintaining contact with the DNA backbone, which is reflected in the IR shifts observed in this study.

The structural insights provided by the IR analysis and machine learning predictions have important implications for understanding the function of EndoIII in DNA repair pathways. The enzyme’s ability to induce conformational changes in the DNA structure is essential for its role in recognizing and excising damaged bases. The observed spectral shifts provide direct evidence of these structural changes, confirming the enzyme’s mode of action at a molecular level.

### Thermodynamic Analysis

Based on the typical assumptions of the dissociation constant (*K*_d_) and enthalpy change (Δ*H*), the following thermodynamic parameters were computed: Δ*G* = 28.52 kJ/mol, Δ*H* = −30.00 kJ/mol, and Δ*S* = −196.39 J/mol·K, showing similar results from the literature.^21,22^ The Gibbs energy for the binding interaction indicates a nonspontaneous process under the assumed conditions. The positive value suggests that the binding interaction between EndoIII and dsDNA is not favorable at the temperature (298 K) and dissociation constant used in this analysis. The binding affinity of EndoIII to dsDNA is a critical factor in determining the Δ*G*. In this case, the *K*_d_ value is an approximation based on typical protein–DNA binding affinities. A *K*_d_ value of 10 μM indicates a relatively weak interaction, which is reflected in positive Δ*G*. A lower *K*_d_ (indicating a stronger binding affinity) would result in a negative Δ*G*. Therefore, this positive Δ*G* suggests that under these conditions, EndoIII may require additional factors or modifications—such as changes in temperature, ionic strength, or the presence of cofactors—to bind to dsDNA effectively.

These thermodynamic parameters align with FTIR observations, confirming the exothermic nature of the EndoIII-dsDNA interaction, which releases heat and stabilizes the complex. FTIR-detected shifts suggest hydrogen bonding and van der Waals forces, consistent with exothermic binding. This likely involves hydrogen bonds between the protein’s active site and the DNA’s phosphate backbone or nitrogenous bases. The negative entropy indicates reduced conformational freedom upon complex formation, supported by FTIR evidence of structural rearrangements such as changes in phosphate backbone vibrations and base-stacking interactions. This is typical of protein–DNA binding, where both molecules adopt rigid conformations. The negative entropy reflects the cost of this reduced flexibility, while the enthalpy highlights stabilizing interactions. Together, these findings demonstrate that the EndoIII-dsDNA binding is governed by a balance of enthalpic and entropic contributions, enabling a specific and structured interaction critical for EndoIII’s role in DNA repair. Studies on protein–DNA interactions have established that negative entropy changes are expected for binding events, attributed to the immobilization of conformational and vibrational degrees of freedom in both the protein and DNA.^21,22^ This aligns with the negative Δ*S* observed in the EndoIII-dsDNA interaction, which likely results from structural rearrangements that create a complementary and stable binding interface. Enthalpy–entropy compensation supports the hypothesis that specific interactions are driven not by a single thermodynamic force but rather by their interplay.

### Correlating Redox Stabilization

The [4Fe–4S] cluster in EndoIII is a redox-active cofactor that undergoes reversible oxidation and reduction, playing an essential role in long-range electron transfer processes that occur during DNA repair.^23^ When bound to DNA, the Fe–S cluster can exchange electrons with the DNA bases, enabling EndoIII to scan for oxidative damage along the DNA strand. The stabilization potential of 150 mV measured in the Fe–S cluster of EndoIII suggests that the cluster is in a relatively stable state when it is bound to DNA. This electrochemical stabilization is crucial for maintaining the structural integrity of the enzyme and enhancing its DNA-binding ability. Electrochemical stabilization directly influences the binding affinity of EndoIII for DNA. Without this stabilization, the Fe–S cluster might undergo conformational fluctuations or lose functionality, reducing its ability to bind to DNA effectively. The redox properties of the Fe–S cluster provide a mechanism for electron transfer between the enzyme and the DNA, further stabilizing the complex.^24^ This process allows EndoIII to access damaged sites in the DNA more efficiently, enhancing its overall repair capabilities.^25^

The thermodynamic parameters of the binding interaction between EndoIII and dsDNA are also influenced by the electrochemical stabilization. Gibbs energy for DNA binding reflects the enthalpic contributions from noncovalent interactions, such as hydrogen bonding and electrostatic interactions, and the entropic contributions from conformational changes. The electrochemical stabilization of the Fe–S cluster adds another layer to this thermodynamic profile by stabilizing the protein’s structure and enhancing the enthalpic component of binding. This stabilization lowers the overall energy of the protein–DNA complex by reducing the energetic cost of maintaining the enzyme’s folded structure. The stabilization potential of 150 mV ensures that the Fe–S cluster remains in a reduced, functional state when interacting with DNA, contributing to a more stable protein–DNA complex.^26^ Moreover, this electrochemical stabilization plays a significant role in facilitating electron transfer between the Fe–S cluster and DNA, further stabilizing the protein–DNA interaction. This redox-driven electron transfer process enhances the scanning function of EndoIII, allowing it to detect and repair damaged bases more effectively. In this context, the electrochemical properties of the Fe–S cluster not only stabilize the enzyme structurally but also enable its functional role in DNA charge transfer and repair.

In the native structure of EndoIII, the Fe–S bond lengths average 2.31 Å with minimal deviation, reflecting a stable cubane-like geometry that supports the structural integrity of the [4Fe4S] cluster. This stable geometry is critical for maintaining the correct orientation of the cysteine ligands, which anchor the cluster and allow for effective electron transfer. However, upon binding to dsDNA, the Fe–S bond lengths decrease to an average of 2.22 Å, suggesting a structural reorganization. This reorganization is driven by electrostatic interactions between the negatively charged phosphate backbone of DNA and the positively charged Fe–S cluster. The structural strain imposed by DNA binding stabilizes the oxidized state (Fe^3+^) of the cluster, which is necessary for redox signaling in DNA charge transfer (DNA-CT). The bond reorganization observed upon DNA binding correlates with shifts in the vibrational modes identified through FTIR spectroscopy. For example, the Fe–S bond vibration, particularly the Fe–S (thiolate) bond, shifts from 363 cm^–1^ in the native state to 353 cm^–1^ upon DNA binding, indicating bond weakening and increased covalency in the oxidized state. This bond weakening supports the notion that the oxidized state of the cluster is stabilized during DNA binding, facilitating the redox communication necessary for DNA repair.

The vibrational frequency shifts observed in the FTIR spectra are consistent with the structural data, where bond length reorganization plays a role in stabilizing the cluster. DFT calculations further validate these findings, predicting similar shifts in vibrational frequencies upon DNA binding. The DFT model shows slight increases in bond lengths for the Fe–S (thiolate) bonds in the DNA-bound state, which aligns with the experimental observations of bond weakening. In addition to bond reorganization, the structural rearrangements observed in the Fe-Cys bond lengths also have thermodynamic implications. The elongation of Fe-Cys bonds in the DNA-bound state suggests that the cysteine ligands experience structural strain, further reducing the system’s entropy. The electrochemical stabilization potential of 150 mV correlates with these structural changes, indicating that the Fe–S bond lengths must be adjusted to accommodate redox changes during DNA-mediated charge transfer. The bond length reorganization observed in the [4Fe4S] cluster is fundamental for maintaining electrochemical stability, ensuring that the cluster remains functional throughout the DNA repair process.

## Conclusions

The relationship between the [4Fe4S] cluster of EndoIII and its interaction with dsDNA is complex, involving electrochemical stabilization, structural changes, and functional modulation through redox control. The combination of IR spectroscopy, DFT calculations, and machine learning reveals that shifts in the Fe–S bond vibrational modes reflect the structural dynamics of the [4Fe4S] cluster during DNA binding. These changes, in turn, affect the enzyme’s DNA-binding affinity and repair function. The electrochemical stabilization potential of 150 mV measured in the [4Fe4S] cluster enhances the enzyme’s ability to bind to DNA, preserving its structural integrity and promoting redox-mediated communication essential for detecting DNA damage. The thermodynamic analysis, which yielded a Gibbs energy change, indicates that while the interaction is exothermic, it is counterbalanced by a negative entropy, showing the structural and conformational adjustments required for EndoIII to achieve its specific and functional DNA-binding state.

Machine learning models trained on IR spectral data predicted key binding parameters, demonstrating the potential of data-driven approaches in elucidating protein–DNA interactions. The combination of machine learning and experimental techniques provides a comprehensive framework for understanding how the [4Fe4S] cluster facilitates DNA repair. Correlating the electrochemical stabilization potential of the cluster with the enzyme’s DNA-binding properties highlights the dual role of the [4Fe4S] cluster in both structural stability and efficient DNA repair. These findings complement previous structural studies that provided static insights into EndoIII’s interaction with damaged DNA.^19^ Crystallographic data revealed that EndoIII bound to DNA with a covalent intermediate at an abasic site, showing how the [4Fe4S] cluster stabilizes DNA through direct interactions. However, redox-driven modulation adds a dynamic layer to this understanding, showing how the oxidized state of the cluster enhances DNA binding and scanning. The dynamic sequence of DNA repair by EndoIII involves several phases: recognition, noncovalent binding, scanning, defect localization, and covalent binding with electron donation and stabilization. Redox activity drives this sequence, directing EndoIII to damaged or mismatched base pairs as part of the base excision repair process.^27^

In summary, the [4Fe4S] cluster plays a dual role in maintaining EndoIII’s structural stability and dynamically modulating its function through redox control. The static structural insights provided by crystallography are complemented by dynamic redox-driven adjustments that influence both DNA binding and scanning. These findings provide a comprehensive landscape of how EndoIII detects, binds, and repairs oxidative DNA damage with redox signaling at the heart of these processes.

## Materials and Methods

### Sample Preparation

Single-stranded DNA (ssDNA) sequences 5′-GTG AGC TAA CGT GTC AGT AC-3′ (20A) and 5′-GTA CTG ACA CGT TAG CTC AC-3′ (20T) were sourced from Integrated DNA Technologies (USA) and stored at −20 °C until needed. To create dsDNA, equimolar amounts of 20A and 20T were mixed to a final concentration of 4 μmol L^–1^. The annealing process involved heating the mixture to 90 °C followed by gradual cooling to room temperature. EndoIII from *E. coli* (≥90%-SDS-Page) was purchased from Sigma-Aldrich and also stored at −20 °C. EndoIII solutions were prepared in 20 mmol L^–1^ sodium phosphate buffer at pH 7.6, supplemented with 0.5 mmol L^–1^ EDTA and 150 mmol L^–1^ NaCl.

### Micro-FTIR Experiments

FTIR spectra and chemical images were collected by using a reflectance mode on a Bruker Vertex 70v FTIR spectrometer coupled with an FTIR microscope (Hyperion 3000, Bruker). The spectrometer was configured to capture spectra at 4 cm^–1^ resolution across the 4000–600 cm^–1^ range, with 100 scans recorded per sample. A focal plane array (FPA) detector, cooled by liquid nitrogen, was used to capture 4096 spectra simultaneously, with each detector element recording data from a spatial area of 2.5 μm. Chemical images were generated by integrating peak intensities for specific vibrational modes. Thin films of EndoIII and the EndoIII/dsDNA complex were prepared by drop-casting 2 μL of each 4 μmol L^–1^ solution onto gold-coated glass slides. The slides were cleaned using acetone, distilled water, and electrochemical cycling in 0.1 mol L^–1^ H_2_SO_4_ between 0.1 and 1.5 V vs Ag/AgCl at a 0.05 V s^–1^ scan rate. After application of the protein solutions, the films were incubated overnight at 4 °C. For the EndoIII/dsDNA complex, equal volumes of both EndoIII and dsDNA were mixed and incubated for 30 min before being deposited onto the substrate. All spectra were presented as difference spectra, with background subtraction applied to remove nonsample contributions.

### Far-IR Experiments

For far-IR analysis, spectra were acquired using the attenuated total reflection (ATR) mode in a vacuum environment on the Bruker Vertex 70v instrument. A diamond crystal served as the internal reflection element and samples of dsDNA and EndoIII were directly placed onto the crystal. The crystal was thoroughly cleaned with 2-propanol and dried between measurements. The EndoIII/dsDNA complex was prepared by combining equimolar concentrations of EndoIII and dsDNA to a final concentration of 2 μmol L^–1^, followed by overnight incubation at 4 °C. Spectra were averaged from 32 interferograms at a 4 cm^–1^ resolution across the 700–100 cm^–1^ range. As with the mid-IR experiments, difference spectra were generated by subtracting the background signals.

### Computational Details

Theoretical simulations of the FTIR spectra were performed by using DFT with the Gaussian 09 software. The model for the [4Fe4S]^2+^ cluster included four ethyl thiolate groups (SCH_3_CH_2_^–^), representing cysteine ligands bound to iron atoms. The total charge of the [4Fe–4S] system was −2. Geometry optimization was based on the coordinates from protein structure 2ABK, with the α-carbons held fixed. LANL2DZ was used as the basis set, and the B3LYP functional was employed. The systems were subjected to a quadratic convergence self-consistent field (SCF) approach, ensuring accurate relaxation without negative frequencies and indicating that the calculations were properly converged. Visualization of the vibrational modes was carried out by using Jmol software.

### Machine Learning for FTIR Data Analysis

We employed machine learning to analyze FTIR spectral data and predict the binding distance between EndoIII and dsDNA. Key features, including peak shifts and intensity changes, were extracted and structured as inputs for a neural network and a Ridge regression model. The neural network (Figure S1) consisted of an input layer (two features), a hidden layer (five neurons), and an output layer predicting the binding distance in angstroms. Spectral features were normalized to reduce bias, and ReLU activation introduced nonlinearity. Ridge regression was utilized for its simplicity and ability to prevent overfitting through regularization (α = 1.0).

Data preprocessing involved baseline correction, smoothing, and peak identification, with qualitative spectral changes converted to numerical values. The data set was split into training (80%) and testing (20%) subsets, with five-fold cross-validation ensuring model robustness.

Binding distances were inferred by correlating FTIR spectral shifts to changes in molecular vibrations, reflecting bonding and electrostatic interactions. Shifts or disappearances of peaks indicated significant interactions, with interaction strength inversely related to the molecular distance. Vibrational frequency changes (Δυ) were analyzed using perturbation theory to link spectral features to bonding and electrostatic forces. The electrostatic interaction energy (*E*) is modeled as inversely proportional to the distance (*r*), in Å. Assuming that the interaction energy is proportional to the frequency shift (Δυ), the distance can be estimated by analyzing shifts in key vibrations, such as phosphate stretching (PO_2_), sugar vibrations, and amide peaks. The actual distance calculation involves determining the absolute frequency shift (|υ–υ_0_|) between the reference peak position (υ_0_) and the shifted peak position (υ) and correlating it to the interaction energy, providing an estimate of the binding distance in eq 1:
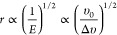
1

This combined methodology provided robust predictions of molecular binding distances while mitigating overfitting risks and ensuring interpretability. Further details are available in the Supporting Information. The Ridge regression model was evaluated using two primary metrics: mean squared error (MSE) and the coefficient of determination (*R*^2^). MSE measures the average squared difference between predicted and actual values, with lower values indicating better model performance. The *R*^2^ value further assesses the proportion of variance explained by the model, providing an additional layer of validation.

### Thermodynamic Analysis

To perform a thermodynamic analysis based on FTIR data and machine learning predictions, we employed mathematical relationships to estimate Gibbs free energy (Δ*G*), enthalpy (Δ*H*), and entropy (Δ*S*) for the interaction between EndoIII and dsDNA. Gibbs energy was used to determine the spontaneity of the binding interaction, where a negative Δ*G* indicates a favorable, spontaneous reaction. The calculation relied on the standard thermodynamic relation Δ*G* = −*RT* ln *K*_d_, with *R* as the universal gas constant (8.314 J/mol K), *T* as the temperature (298 K), and *K*_d_ as the dissociation constant. Although direct binding data were unavailable, *K*_d_ was approximated from the relative intensity changes observed in the FTIR spectra. Spectral shifts and intensity variations in key peaks, such as those corresponding to O–P–O bending, sugar–phosphate vibrations, and CH_2_ wagging, were used as proxies for interaction strength. Enthalpy change was inferred to represent the heat released or absorbed during binding. Although FTIR does not directly measure Δ*H*, shifts in peaks associated with NH and OH stretches suggest hydrogen bond formation between EndoIII and dsDNA. Hydrogen bonding is commonly associated with a negative Δ*H*, indicating heat release upon formation. Drawing from literature values for similar interactions, Δ*H* was estimated at −30 kJ/mol, a value consistent with typical protein–DNA interactions,^21,22^ which range from −20 to −50 kJ/mol. Entropy change (Δ*S*) was estimated by using the Gibbs free energy equation, Δ*G* = Δ*H* – *T*Δ*S*, with Δ*G* and Δ*H* values serving as inputs. Protein–DNA binding typically reduces entropy due to increased molecular order, though solvent reorganization and conformational adjustments may counterbalance this effect.

## Supporting Information Available

Neural network model with FTIR spectral features as input; training used MSE loss, gradient descent, and cross-validation; FTIR analysis covered key spectral shifts; Ridge regression predicted binding distance with normalization; vibrational shifts; neural network architecture; interaction distances; FTIR peak summary; and vibrational shift calculations (PDF)

## Accession Codes

Endonuclease III (EndoIII) enzyme from *Escherichia coli*, UnitProt Accession number P0AB83.

## Abbreviations

EndoIII: Endonuclease III
BER: base excision repair
DNA-CT: DNA-mediate charge transfer
dsDNA: double-stranded DNA
IR: infrared
DFT: density functional theory
MIR: mid-infrared
Far-IR: far-infrared
ATR: attenuated total reflection
FTIR: Fourier transform infrared
PDB: protein data bank
MSE: mean squared error
