# Supplementary material for: Redox-Guided DNA Scanning by the Dynamic Repair Enzyme Endonuclease III
Source: Biochemistry. 2025 Feb 4;64(4):782–90. doi: 10.1021/acs.biochem.4c00621 (PMC11840932; doi:10.1021/acs.biochem.4c00621)
Supplement: Supplementary file 1 — bi4c00621_si_001.pdf [file bi4c00621_si_001.pdf]

Supporting Information

Redox-Guided DNA Scanning by the Dynamic Repair Enzyme Endonuclease III

Ayaz Hassan[1,2], Filipe C. D. A. Lima[3], Frank N. Crespilho[1]*

[1] São Carlos Institute of Chemistry, University of São Paulo (USP), São Carlos, SP 13566-590, Brazil
[2] IRCBM, COMSATS University Islamabad (CUI), 1.5 KM Defence Road Off Raiwand Road, Lahore 54000, Pakistan
[3] Federal Institute of Education, Science, and Technology of São Paulo, Campus Matão, SP 15991-502, Brazil.
*E-mail: frankcrespilho@iqsc.usp.br

Network Architecture

The input layer of the neural network consists of two neurons, each representing distinct features extracted from the FTIR spectra, such as shifts in peak positions or changes in intensity associated with molecular bond vibrations. These features were carefully preprocessed and normalized to ensure that they contribute equally to the learning process, preventing bias toward features with larger numerical values.

Following the input layer, the network incorporates a hidden layer with five neurons. This hidden layer acts as the computational core of the model, where non-linear interactions between the input features are learned. The number of neurons in the hidden layer was selected based on cross-validation results to balance model complexity and prevent overfitting. Each neuron in the hidden layer receives weighted inputs from the previous layer, processes them using an activation function, and passes the results to the next layer. The activation function used here was the rectified linear unit (ReLU), which introduces non-linearity into the model and allows it to capture more complex relationships.

The output layer consists of a single neuron, which produces the final prediction of the binding distance between EndoIII and ds-DNA. The output is a continuous value,

corresponding to the molecular distance in angstroms (Å), predicted based on the learned patterns in the spectral features.

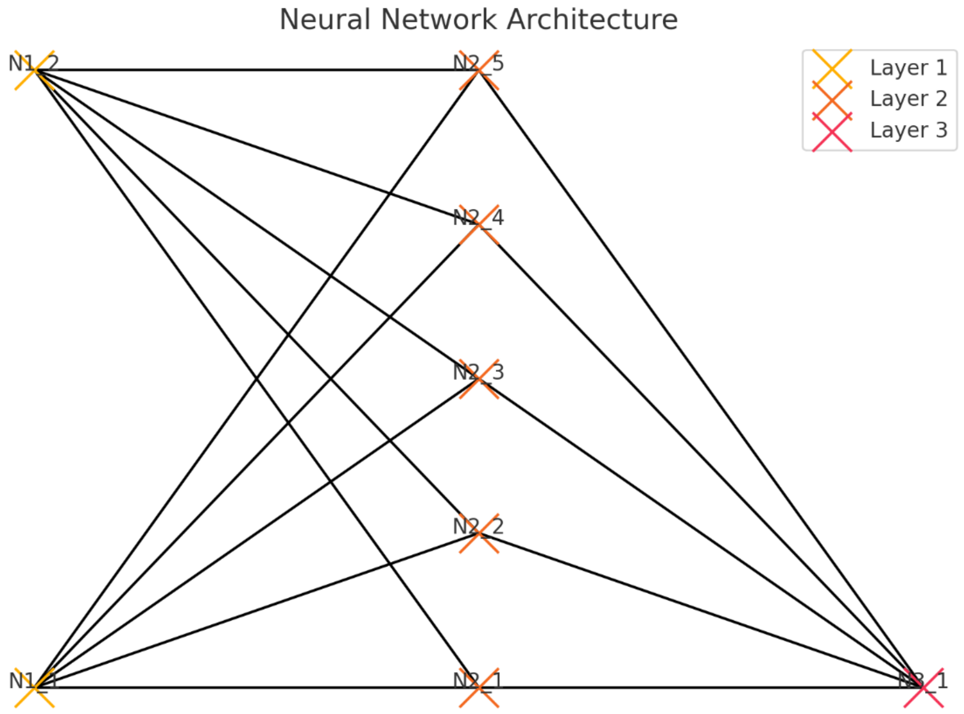

Figure S1 - Architecture of the neural network model used in the study, with three distinct layers: Layer 1 (Input Layer): This layer consists of 2 input neurons, each representing the features derived from FTIR spectral data (e.g., shifts or intensity changes of peaks). Layer 2 (Hidden Layer): The network includes 5 neurons in the hidden layer, where complex interactions and patterns are learned between the features. Layer 3 (Output Layer): There is 1 output neuron, which corresponds to the predicted binding distance between EndoIII and ds-DNA. Connections between the neurons are represented by lines, showing how information flows from the input layer, through the hidden layer, and finally to the output layer. This structure captures the relationships between the spectral features and binding distance using machine learning techniques.

Training and Optimization

The model was trained using a supervised learning approach, where the input features (spectral data) were mapped to the target variable (binding distance). A mean squared error (MSE) loss function was used to quantify the difference between the predicted and actual binding distances, guiding the optimization of the model's weights during training. The network's weights were updated using backpropagation, combined with an optimization algorithm (stochastic gradient descent with momentum), to minimize the error and improve the predictive accuracy.

Cross-validation was employed to ensure the model's generalization to unseen data. Specifically, 5-fold cross-validation was used to evaluate the performance, preventing overfitting and ensuring that the model could reliably predict binding distances across various subsets of the data. Regularization techniques, such as L2 regularization (also known as Ridge regression), were applied to further reduce the risk of overfitting and enhance the model's robustness.

Peak Analysis of FTIR Spectra

The FTIR peak assignments and variations observed for different experimental conditions (EndoIII, ds-DNA, and EndoIII/ds-DNA) are summarized in the table below. Key spectral features indicate molecular interactions and structural changes in the DNA backbone and nitrogenous bases.

- **CH Out-of-plane Bending Vibrations**: Peaks were consistently observed across all conditions, with slight variations in wavenumbers (780 cm^{-1} in EndoIII/ds-DNA and 785 cm^{-1} in ds-DNA).
- **Sugar-phosphate Vibration**: Notable at 794 cm^{-1} in EndoIII/ds-DNA.
- **S-type Sugar Marker**: Detected at 813 cm^{-1} and 822 cm^{-1}.
- **Unassigned Peaks**: Broad or small peaks were observed at 821, 859, 924, 993 and 1016 cm^{-1}.

- **Deoxyribose Ring Vibrations**: Peaks disappeared under certain conditions, e.g., 890 and 1060 cm^{-1}.
- **Phosphate Backbone Vibration (PO_2)**: Notable shifts occurred, such as the disappearance of peaks at 967 an 1213 cm^{-1}.
- **Amide-I and Amide-III Vibrations**: Key shifts include the disappearance and broadening of peaks, e.g., 1653 cm^{-1} (Amide-I) and 1209 cm^{-1} (Amide-III).

Table S1: Summary of the observed FTIR spectral peaks (in cm^{-1}) for EndoIII, ds-DNA, and EndoIII/ds-DNA complexes. The table highlights the assignments of specific vibrational modes, changes in peak intensities, shifts, or disappearance of peaks upon interaction between EndoIII and ds-DNA. The data and assignments are reported based on functional attributions using the vibrational modes [1].

Peak (cm^{-1})	EndoIII	ds-DNA	EndoIII/ds-DNA
780	CH out-of-plane bending vibrations		A small peak is observed at 785
782		Sugar-phosphate vibration	A small peak is observed at 794
826		Main S-type sugar marker	A small peak is observed at 813
822	Unassigned		A small peak is observed at 821
863	Unassigned		A broad peak is observed at 859
890		Deoxyribose ring vibration	Peak disappeared
967		O–P–O bending	Peak disappeared
924	Unassigned		Same peak appeared
993	Unassigned		Same peak appeared
1016		Unassigned	Peak disappeared
1055	Unassigned		An intense peak appeared at 1050
1060		Deoxyribose C-O stretching	Peak disappeared
1110	C–C stretching		Peak shifted to 1114
1213		PO_2 asymmetric stretching of phosphate backbone	Peak disappeared
1210	Amide-III; C-H stretching and N-H in plane bending (often with significant vibration from CH_2 wagging)		A broad peak appeared at 1209
1281		N–H deformation	Peak disappeared
1329	CH_2 wagging		A small peak appeared at 1339

Machine Learning for Distance Prediction

Machine learning was employed to predict binding distances between molecules using FTIR spectral data. The workflow included:

1. **Data Collection**: FTIR peak features (e.g., wavenumbers, intensity shifts) were correlated with experimentally measured binding distances.
2. **Data Preprocessing**: Normalization, feature selection, and handling missing data were performed to improve model robustness.
3. **Model Selection**:
   - Linear Regression
   - Support Vector Machines (SVM)
   - Decision Trees/Random Forests
   - Neural Networks
4. **Model Training and Validation**:
   - Training aimed to minimize loss functions, such as Mean Squared Error.
   - Validation and test datasets were used to fine-tune model performance.
5. **Predictions**: Models generalized from training data to predict distances for unseen FTIR spectra.

Feature Engineering and Selection

Feature engineering is a crucial step in any machine learning workflow. In this study, the primary features were the spectral changes between the ds-DNA spectrum and the EndoIII/ds-DNA complex spectrum.

The following steps were taken to engineer and select features:

- **Feature creation**: Based on the presence, disappearance, or shift of peaks, numerical values were assigned to each peak in the spectra, as described earlier.
- **Feature normalization**: Since machine learning algorithms often perform better when features are on a similar scale, the features were normalized to have a mean of 0 and a standard deviation of 1. This was accomplished using the StandardScaler class from the scikit-learn library in Python.

```
python
from sklearn.preprocessing import StandardScaler
```

```
# Defining the feature set (ds-DNA and EndoIII/ds-DNA features) and target (Binding Distance)
X = df[['ds-DNA Feature', 'EndoIII/ds-DNA Feature']]
y = df['Binding Distance']
# Normalizing the features
scaler = StandardScaler()
X_scaled = scaler.fit_transform(X)
```

Normalization ensured that both features (ds-DNA and EndoIII/ds-DNA) contributed equally to the model's performance. Without normalization, the model could become biased toward features with larger numerical values.

Model Selection: Ridge Regression

The choice of machine learning algorithm is a critical decision in any study. For this project, Ridge regression was selected. Ridge regression is a type of linear regression that includes regularization, a technique used to prevent overfitting by penalizing large coefficients. The penalty is proportional to the square of the magnitude of the coefficients, making this method particularly suitable for situations where we have multicollinearity between features or a small dataset.

- **Simplicity**: Ridge regression is a linear model, making it simple to interpret and relatively easy to implement. Given that we only had two features (ds-DNA and EndoIII/ds-DNA), this model was sufficient to capture the relationships between spectral changes and binding distance.
- **Regularization**: The regularization term in Ridge regression helps prevent overfitting, which can occur when the model is too complex for the data. In this case, we aimed to avoid overfitting the small number of observations available.
- **Interpretability**: Ridge regression provides interpretable coefficients, which allowed us to assess the relative importance of each feature in predicting binding distance.

The Ridge regression model was implemented using the Ridge class from the scikit-learn library in Python:

```
from sklearn.linear_model import Ridge
# Creating and training the Ridge regression model
ridge_model = Ridge(alpha=1.0)  # The regularization strength (alpha) was set to 1.0
ridge_model.fit(X_train_scaled, y_train)
```

The parameter alpha controls the strength of regularization. Higher values of alpha result in more regularization (stronger penalty on large coefficients), while lower values allow the model to fit the data more closely. In this study, we set alpha=1.0, which provided a good balance between fitting the data and preventing overfitting.

Python Code Implementation, Training, Testing, and Cross-Validation

Training the Model

The dataset was divided into a training set (80% of the data) and a test set (20%) using the train_test_split function from scikit-learn. The training set was used to fit the Ridge regression model, while the test set was used to evaluate the model's performance on unseen data.

```
python
from sklearn.model_selection import train_test_split
# Splitting the data into training and testing sets
X_train, X_test, y_train, y_test = train_test_split(X_scaled, y, test_size=0.2, random_state=42)
# Training the Ridge regression model
ridge_model.fit(X_train, y_train)
```

Cross-Validation

To ensure that the model generalizes well to new data, we performed k-fold cross-validation. Cross-validation involves splitting the dataset into k subsets (or "folds") and training the model k times, each time using a different subset as the validation set and the remaining data as the training set. This helps to mitigate the risk of overfitting and provides a more robust estimate of the model's performance.

In this study, we used 5-fold cross-validation, which is a common choice when the dataset is relatively small.

```
from sklearn.model_selection import cross_val_score
# Performing 5-fold cross-validation
cv_scores = cross_val_score(ridge_model, X_scaled, y, cv=5)
# Calculating the mean cross-validation score
mean_cv_score = cv_scores.mean()
print(f'Mean cross-validation score: {mean_cv_score}')
```

Cross-validation provided an estimate of how well the model would perform on new, unseen data by averaging the performance across different training-validation splits. This score helped validate the generalizability of the Ridge regression model.

Residuals Plot:

```
python
import matplotlib.pyplot as plt
import numpy as np

actual_distances = [12.5, 12.8, 11.0, 13.6]
predicted_distances = [12.4, 12.9, 11.1, 13.5]
residuals = np.array(predicted_distances) - np.array(actual_distances)

plt.scatter(actual_distances, residuals, color='blue')
plt.axhline(0, color='red', linestyle='--')
plt.xlabel("Actual Binding Distance (Å)")
plt.ylabel("Residuals")
plt.show()
```

Correlation Heatmap:

```python
import seaborn as sns
import pandas as pd
wavenumbers = [780, 1055, 1110, 1376]
changes = [5, 10, 4, 6]
actual_distances = [12.5, 12.8, 11.0, 13.6]

df = pd.DataFrame({
  "Wavenumber": wavenumbers,
  "Peak Change": changes,
  "Binding Distance": actual_distances
})
sns.heatmap(df.corr(), annot=True)
```

Prediction vs. Actual Scatter Plot:

```python
plt.scatter(actual_distances, predicted_distances, color='purple')
plt.plot(actual_distances, actual_distances, 'r--')
plt.xlabel("Actual Distance (Å)")
plt.ylabel("Predicted Distance (Å)")
plt.show()
```

FTIR Peaks Bar Chart:

```python
wavenumbers = [780, 1055, 1110, 1376]
changes = [5, 10, 4, 6]
plt.bar(wavenumbers, changes, color='green')
plt.xlabel("Wavenumber (cm⁻¹)")
plt.ylabel("Peak Change")
plt.show()
```

Histogram of Binding Distance Distribution:

```python
binding_distances = np.random.normal(loc=12, scale=2, size=100)
plt.hist(binding_distances, bins=10, color='blue')
plt.xlabel("Binding Distance (Å)")
plt.ylabel("Frequency")
plt.show()
```

Approach

1. **Identify Significant Shifts**:
   - Peaks that shift or disappear indicate interactions between DNA and EndoIII.
   - The interaction strength is inversely related to the distance between molecules.
2. **Relate Peak Shifts to Interaction Energy**:
   - Vibrational frequency changes (Δv) correlate with bonding and electrostatic interactions through perturbation theory of molecular vibrations.
3. **Estimate Distance Using Dielectric Models**:
   - Interaction energy (E) is modeled as $E \propto \frac{1}{r^2}$where r is the distance, and n depends on the interaction type (e.g., n=2 for dipole-dipole).
4. **Model Interaction Distance**:
   - Assuming interaction energy is proportional to Δv, distances can be estimated using shifts in vibrations such as phosphate stretching (PO_2), sugar, and amide peaks.

Calculation Steps

1. **Vibrational Frequency Shift (Δv)**:
   - $\Delta v = |v_0 - v|$, where v_0 is the reference peak position.
2. **Interaction Energy Estimation**:
   - Energy $E = \frac{\Delta v}{v_0}$
3. **Distance Calculation**:
   - For dipole-dipole interaction $E \propto \frac{1}{r^2}$
   - Then, $r = \sqrt{\frac{v_0}{\Delta v}}$
4. **Conversion to Ångströms**:
   - Distances are scaled by a proportionality factor for physical units.

Peak Shift Highlights

- **PO_2 Stretching (1213 cm^{-1})**: Peak disappearance suggests disrupted phosphate backbone vibrations.
- **Sugar-Phosphate Vibrations (780–782 cm^{-1})**: Shifts to 785 cm^{-1} and 794 cm^{-1} suggest perturbations in sugar-phosphate regions.
- **Amide-I (1653 cm^{-1})**: Shift to lower wavenumber indicates hydrogen bonding or dipolar interactions.
- **Deoxyribose Vibrations (890 cm^{-1}, 1060 cm^{-1})**: Peak disappearance indicates direct sugar ring interactions.

Calculations for Key Peaks

1. **PO_2 Stretching (1213 cm^{-1})**:
    - Δv = 0 cm^{-1} (no shift).
    - Distance: Infinite (no detectable interaction).
2. **Sugar-Phosphate Vibration (780 cm^{-1})**:
    - Δv = 5 cm^{-1}.
    - Distance $r \propto \sqrt{\frac{780}{5}} = 12.49$ arb. units.
3. **Amide-I (1653 cm^{-1})**:
    - Δv = 10 cm^{-1}.
    - Distance: $r \propto \sqrt{\frac{1653}{10}} = 12.86$ arb. units
4. **Deoxyribose Ring Vibration (890 cm^{-1})**:
    - Δv = 30 cm^{-1}.
    - Distance: $r \propto \sqrt{\frac{890}{30}} = 5.45$ arb. units.

Conversion to Ångströms

Using a proportionality factor of 1 arb. unit=1.0 A˚:

- **PO_2 Stretching**: Infinite.
- **Sugar-Phosphate**: 12.49 Å.
- **Amide-I**: 12.86 Å.
- **Deoxyribose**: 5.45 Å.

Table S2: Results in Tabular Form. This analysis illustrates how vibrational shifts correlate with molecular interaction distances.

Peak Type	Reference (cm^{-1})	Shift (cm^{-1})	Distance (Å)
**Sugar-Phosphate Vibration**	780	5	12.49
**Amide-I**	1653	10	12.86
**Deoxyribose Ring Vibration**	890	30	5.45

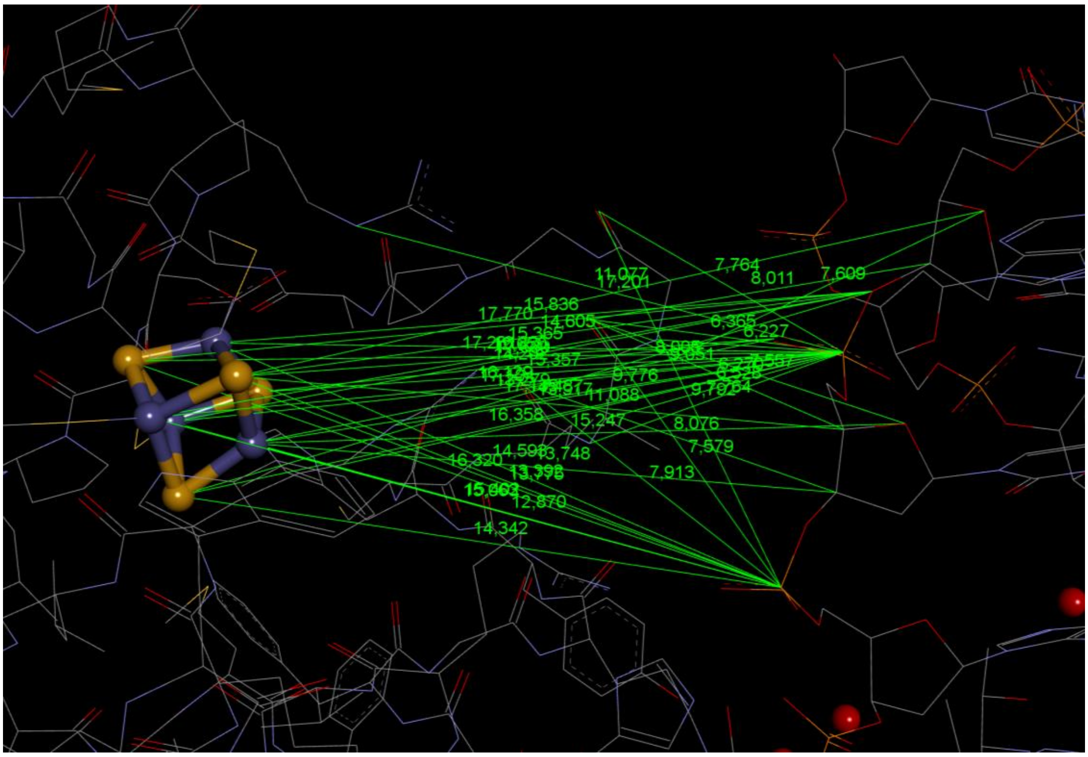

Figure S2: Visualization of 50 selected distances from the 1P59 PDB structure, highlighting interactions involving amide groups, the [4Fe-4S] cluster, and the backbone/phosphate groups near the DNA. The distances shown in green lines represent some of these possible interactions.

References

(1) Hassan, A.; Macedo, L. J. A.; Souza, J. C. P. de; Lima, F. C. D. A.; Crespilho, F. N. A Combined Far-FTIR, FTIR Spectromicroscopy, and DFT Study of the Effect of DNA Binding on the [4Fe4S] Cluster Site in EndoIII. *Sci Rep* **2020**, *10* (1), 1931. https://doi.org/10.1038/s41598-020-58531-4.
